# Simulation Tool for the Analysis of Cooperative Localization Algorithms for Wireless Sensor Networks

**DOI:** 10.3390/s19132866

**Published:** 2019-06-27

**Authors:** Mario L. Ruz, Juan Garrido, Jorge Jiménez, Reino Virrankoski, Francisco Vázquez

**Affiliations:** 1 Department of Mechanical Engineering, University of Cordoba, Campus de Rabanales, 14071 Cordoba, Spain; 2 Department of Computer Science and Numerical Analysis, University of Cordoba, Campus de Rabanales, 14071 Cordoba, Spain; 3 Department of Communications and Networking, School of Electrical Engineering, Aalto University, FI-00076 Aalto, Finland

**Keywords:** algorithms, indoor localization, cooperative localization, industrial safety, IoT, LoT

## Abstract

Within the context of the Internet of Things (IoT) and the Location of Things (LoT) service, this paper presents an interactive tool to quantitatively analyze the performance of cooperative localization techniques for wireless sensor networks (WSNs). In these types of algorithms, nodes help each other determine their location based on some signal metrics such as time of arrival (TOA), received signal strength (RSS), or a fusion of them. The developed tool is intended to provide researchers and designers a fast way to measure the performance of localization algorithms considering specific network topologies. Using TOA or RSS models, the Crámer-Rao lower bound (CRLB) has been implemented within the tool. This lower bound can be used as a benchmark for testing a particular algorithm for specific channel characteristics and WSN topology, which allows determination if the necessary accuracy for a specific application is possible. Furthermore, the tool allows us to consider independent characteristics for each node in the WSN. This feature allows the avoidance of the typical “disk graph model,” which is usually applied to test cooperative localization algorithms. The tool allows us to run Monte-Carlo simulations and generate statistical reports. A set of basic illustrative examples are described comparing the performance of different localization algorithms and showing the capabilities of the presented tool.

## 1. Introduction

Internet of Things (IoT) is a paradigm intended as a network of a very large number of sensor nodes deployed over physical space. These sensors form a wireless sensor network (WSN) and have capabilities to monitor the physical environment and collect and report data for a specific application. WSNs can enhance the capabilities in a wide variety of applications where the location information plays an important role [1]. Some examples include military services, search and rescue operations, tracking of objects and people, logistics, etc. With the emerging new technologies enabling accurate positioning, the IoT opens an important dimension called Location of Things (LoT), where the position of nodes plays an important role and the cooperative localization algorithms have the potential to open the way to new and revolutionary applications [2]. For example, within the context of industrial safety, reducing the risk of possible accidents is a key aspect. The integration of advanced positioning and sensor communications can be considered an enabling technology for “augmented” safety. WSNs can provide new capabilities for improved safety for several military and civilian applications. Triggering alarms and/or stopping machines when human intrusions into dangerous areas are detected could be achieved by knowing the position of the workers [3]. Localization of personnel in underground operations, such as those carried out in the mining industry, can be extremely useful in case of an accident. A wide variety of such applications could be enabled with the use of WSNs [2,4,5]. Additionally, the robustness of the localization system must be considered. Parameters such as accuracy of the positioning in different conditions, immunity to multi-path fading, power consumption of the sensors, the network topology, and density are critical application-specific factors to be considered.

Simulating the performance of a localization algorithm in a specific scenario can help designers define the minimum sensor requirements for a set of positioning applications. In this work, we present an interactive simulation tool, named wsnLocalize, for testing different localization algorithms in WSNs, specifically those that belong to cooperative localization techniques, which allow sensors that are not in range of known-location devices (also called anchors) to be located by means of the estimation of pairwise distances. The distance estimation process is carried out by a data fusion process, where the pairwise distances are collected and integrated in a centralized or distributed way. The developed tool is intended to compare and evaluate such methods, providing researchers and designers a fast way to measure the performance of different localization algorithms in a modelled scenario. The interactivity provided allows the user to instantaneously see the effects on the localization performance by simply changing node positions. In the same way, a different set of parameters can be changed, such as the noise added to the distance measurements, the node range, or the probability of failure. The tool exploits the Monte-Carlo method, which allows the generation of statistical reports for a specific localization algorithm and a concrete network configuration.

The rest of the paper is organized as follows. The location estimation problem is formalized in Section 2, including a brief literature review about cooperative localization algorithms and applications. In addition, the Cramér-Rao lower bound (CRLB) is presented as benchmark tool for testing the performance of a specific localization algorithm. In Section 3, a description regarding the use of the tool wsnLocalize is described, by outlining its main features. Section 4 presents a set of basic illustrative examples results comparing some state-of-the-art algorithms, which also considers different network topologies. Conclusions are summarized in Section 5. Lastly, an appendix is included containing the main stress functions used for the performance analysis of the localization algorithms.

## 2. Background

### 2.1. Problem Statement and Related Work

The purpose of a cooperative localization algorithm is to estimate the coordinates of *n* of the sensors, called non-anchor nodes or blind nodes, given the coordinates of *m* of the sensors, called anchor nodes. Thus, the total number of nodes in the WSN is defined as *N = n + m*. Considering the two dimensional localization problem [6], a total of 2*n* unknown-location node parameters are estimated, which are defined by $\theta ={\left[\theta_{x},\theta_{y}\right]}^{T}$, where

(1) $$\theta_{x}=\left[x_{1},x_{2},\ldots ,x_{n}\right],\theta_{y}=\left[y_{1},y_{2},\ldots ,y_{n}\right].$$

The term “cooperative” means that nodes help each other determine their locations. The anchor nodes coordinates, whose absolute coordinates are known a priori, are defined as $\left[x_{n+1},x_{n+2},\ldots ,x_{n+m,}\;y_{n+1},y_{n+2},\ldots ,y_{n+m}\right]$. Two sensors with a communication link between them may be able to measure the distance between them and use it for localization [7]. The set of pairwise measurements is defined as $\left\{X_{i,j}\right\}$, where $X_{i,j}$ represents the measurement between the node i and node j from the network. This measurement can be any physical reading that indicates distance or relative positioning, i.e., time-of-arrival (TOA), time-difference-of-arrival (TDOA), angle-of-arrival (AOA), received-signal-strength (RSS), or a combination of them [5,6]. The connectivity (whether two nodes can communicate) can also be used for localization purposes. Ultra-wideband (UWB) technology is a promising solution due to its potential for high accurate localization [3,5,8,9,10], specifically with time-based positioning techniques due to the high time resolution of UWB signals. Regardless of the technology employed, the previously mentioned signal metrics are susceptible to different sources of errors that must be considered to carry out the location task with high accuracy. The accuracy of localization depends on the reliability of the information exchanged between devices in the network. Localization algorithms are sensitive to interferences, environmental effects, multipath, noise, obstructions, and diverse sources that affect the measurement phase and degrade the localization task [8]. There is a growing body of literature to deal with these pitfalls and challenges. For example, accurate clock synchronization is required when TOA measurements are employed. Within the rich literature on synchronization techniques in WSNs, recent developments treat this challenge in a collaborative fashion to mitigate the effects of imperfect clocks [11,12]. Another direction of research is the development of methods focused on the detection of malfunctioning or malicious nodes that inject false information in the network. Detection of these outliers and malfunctioning nodes is of critical importance for preserving the accuracy of the localization task. Cooperative methods to deal with this problem are discussed in References [13,14].

**Figure 1:**
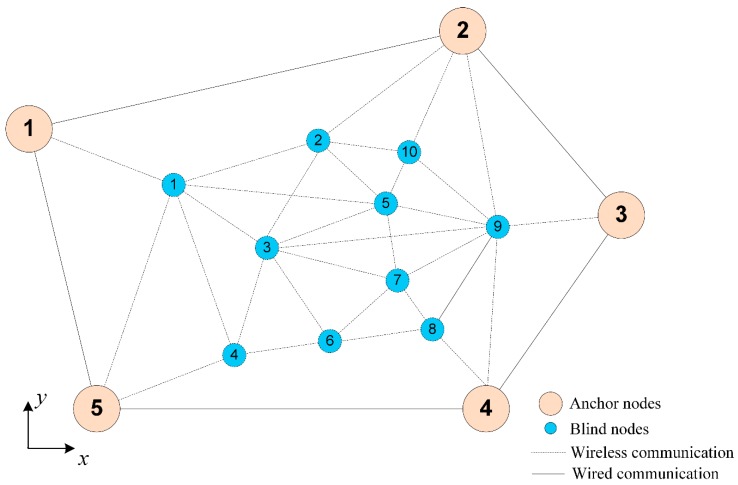
General scheme of a wireless sensor networks (WSN) represented by a graph.

As mentioned above, it is well known that, in realistic deployments, obstacles and large node separations render the collection of all pairwise distances infeasible [15] and the cooperative localization task can be severely degraded [16]. Thus, it is assumed that not all pairwise distance measurements are known (i.e., internode measurements). An important issue in the node localization problem is to provide an efficient algorithm of the distance matrix reconstruction even in the presence of noise and sensor node failures [15,17,18]. For example, given a WSN of N nodes, where N=n+m, there are N·(N−1)/2 distance pairwise measurements, but the knowledge of these distances is partial with noise. A first phase of cooperative localization algorithms tries to infer the unknown distances [15]. As described in Reference [7], the communication link between the sensors can be modeled as a sensor graph. This sensor graph, together with pairwise distances measured along its edges, is called the measurement graph. An example is shown in Figure 1, where there are some nodes that cannot communicate with each other directly, such as node 2 with 9, or 1 and 10. In the first step, cooperative localization algorithms try to estimate the missing distances from the known distances [19,20,21]. Based on this information, cooperative localization techniques try to estimate simultaneously the node coordinates, i.e., the parameter vector θ. Factors such as accuracy of the type of measurement made and transmitted between the nodes of the network will affect the global performance of the localization algorithm. There will be a non-deterministic (random) component in the measurement. In realistic scenarios, the measurement graph is noisy and sparse, which means that only a small amount of unreliable information is available [7].

**Figure 2:**
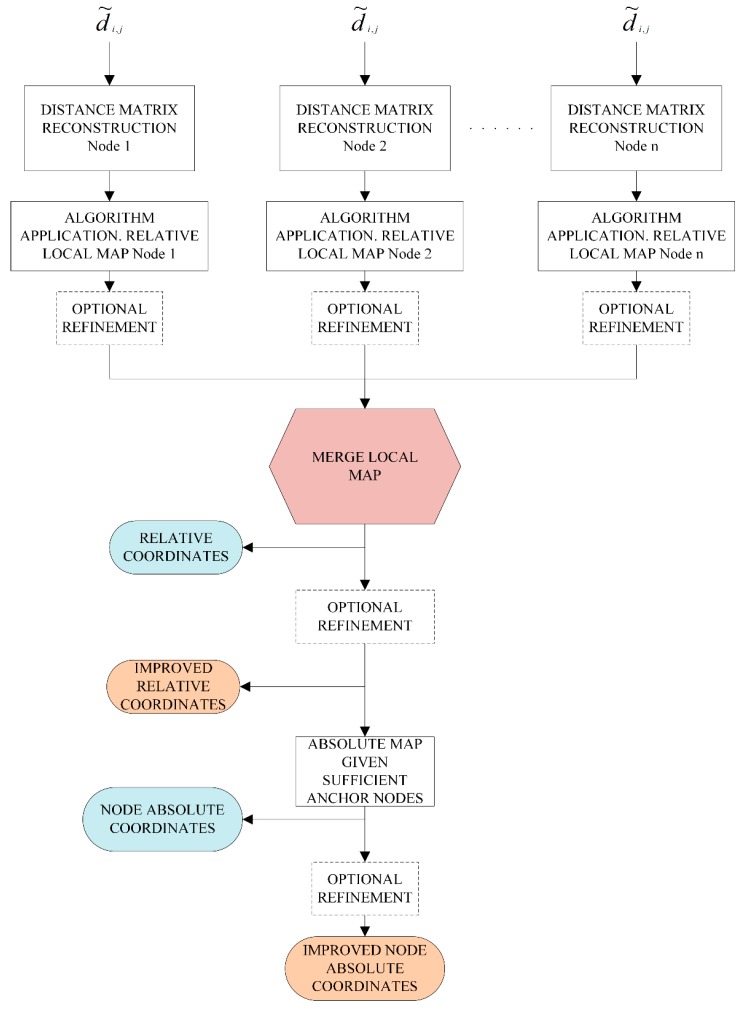
General cooperative localization steps.

From a general point of view, Figure 2 summarizes the main steps of cooperative localization algorithms. In the first step, once the pairwise distances have been estimated (measurement phase), the missing distances must be computed. As in Reference [7], in this work, it is assumed that a WSN is modeled by a graph such that for every edge (i,j) a distance estimation ${\tilde{d}}_{i,j}$ is known. The environment, the metrics (AOA, RSS, TOA) and the technology employed will affect the accuracy of ${\tilde{d}}_{i,j}$. In addition, depending on the algorithm, this step can be carried out in a distributed fashion, which is appropriate for large-scale WSNs [20]. Thus, distributed algorithms will compute relative local maps that will be merged together to build a global relative map of the graph, providing an “embedding” that realizes the pairwise estimated distances. If enough anchor nodes are available, in a second step (location phase), the relative map will be transformed to an absolute map. Postprocessing refinement stages can be executed, as shown in Figure 2, which improves the solution at the expense of a higher computational cost.

An overview of cooperative localization algorithms is given in References [5,8,14]. One of the well-known techniques used for cooperative localization is multi-dimensional scaling (MDS). There are many types of MDS techniques developed methods. They can be classified according to the nature of the MDS model (metric or non-metric), the number of matrices used to represent pairwise distances, or if the approach is deterministic or probabilistic. Some examples are MDSMAP-(C), MDS-MAP(D), or distributed weighted multidimensional scaling (dwMDS). Further details and precise mathematical presentation of these methods can be found in References [8,20,21,22,23,24,25]. It is important to note that if all the noise-free pairwise distances were known, MDS techniques would provide an exact solution. However, this situation changes dramatically when only a subset of pairwise distances is available, and iterative methods must be employed to refine the solution. 

Distributed localization algorithms are required to be practical and scalable in large networks. Based on the cooperative approach, powerful localization algorithms have been proposed in the last decade, such as ARAP (as-rigid-as-possible) [7], the mentioned dwMDS [25], and SPAWN (sum-product algorithm over a wireless network) [8]. The latter one provides excellent performance with low latency. Nevertheless, its main drawbacks are its high computational complexity and large amount of network traffic. New methods based on the SPAWN algorithm have been proposed with allegedly lower computational complexity [26,27,28].

Additionally, hybrid and cooperative mobile positioning algorithms have emerged as a new stream of wireless location, such as HSPAWN, the hybrid version of SPAWN, which fuses information from satellites and WSNs [2]. A review on recent techniques and concepts used to improve localization is provided in [5], including a comprehensive list of localization challenges in next generation 5G networks.

As mentioned, estimated distances are measured from a physical medium that introduces errors. Generally, these measurements are impacted by static, time-varying errors, and also environment-dependent errors [6]. Thus, the measurements carried out are characterized as random variables. By means of statistical models based on the previously mentioned measurements, pairwise distances can be estimated. The key to develop a precise cooperative localization system is to accurately represent the degrading effects of the channel in which the pairwise measurements are made. The different statistical characterizations for RSS, TOA, and AOA measurements are contrasted both analytically as empirical measurements through different investigations [6]. As an example of an RSS model, which is defined as the voltage measured by a receiver’s signal strength indicator (RSSI) circuit, the ensemble mean power at distance d is typically modeled as follows [4].

(2) $$\bar{P}\left(d\right)=P_{0}-10n_{p}\log_{10}\left(\frac{d}{d_{o}}\right),$$

where $P_{0}$ is the received power (dBm) at a short reference distance $d_{o}$, and $n_{p}$ is the path-loss exponent, which is typically between two and four [6]. Given the true separation between nodes i and j:

(3) $$d_{i,j}=\sqrt{{\left(x_{i}-x_{j}\right)}^{2}+{\left(y_{i}-y_{j}\right)}^{2}}.$$

The estimated distance, i.e., the range between nodes i and j can be estimated as follows.

(4) $${\tilde{d}}_{i,j}=\frac{d_{o}}{C}10^{\frac{d_{o}-P_{i,j}}{10n_{p}}},$$

where C is a multiplicative bias factor and is approximately equal to C≈ 1.2 for typical channels [4].

As mentioned, under perfect and complete information, the cooperative localization algorithms would provide the true coordinates of the blind nodes. However, this situation is not realistic and it is the main reason of the model developments for the distance estimates. The reader is referred to References [4,6,8] and the references therein offer more detailed information about measurement-based statistical models for a range.

### 2.2. The Cramér-Rao Lower Bound (CRLB)

The Cramér-Rao bound provides a means for calculating a lower bound on the covariance of any unbiased location estimator [4,6,29]. Such a lower bound can be used as a benchmark for testing a particular algorithm and allow determination if the necessary accuracy for a specific application is possible. The CRLB has been implemented in wsnLocalize, providing researchers insight about the behavior of a specific localization algorithm given a specific network topology (e.g., random placement, square grid, etc.). As explained in [6], the CRB can be used as a guideline, providing the best possible accuracy under a set of specific network features. Its calculation only requires the statistical model of the random measurements, i.e., f(X|θ), where X represents the random variable of measurements and θ the 2*n* unknown-location node parameters to be estimated and specified in Equation (1). Any unbiased estimator must satisfy the following equation [6].

(5) $$cov\left(\hat{\theta }\right)\geq {\left\{-E\left[\nabla_{\theta }{\left(\nabla_{\theta }lnf\left(X;\theta \right)\right)}^{T}\right]\right\}}^{-1},$$

where $cov\left(\hat{\theta }\right)$ represents the covariance of the estimator, E[·] indicates the expected value, $\nabla_{\theta }$ is the gradient operator with respect to the vector θ, and superscript *T* indicates transpose. Both Reference [6] and Reference [29] derive and provide the expressions as well as a detailed explanation of the CRLB for RSSI, TOA, and only-connectivity measurements. The CRLB estimator covariance is a function of the sensor geometry, the number of anchors and blind nodes, measurement type, network connectivity, and channel parameters. The contributions of the CRLB limit should be clear. On one hand, the CRLB provides a minimum level or "best possible case" for the estimation of one or more parameters, which, in the particular case of localization, are the x and y node coordinates. On the other hand, the CRLB provides the characteristics that should be modified to improve the achievable accuracies in the estimation of the parameters under a set of specific conditions. Thus, the CRLB provides a unique mechanism to answer questions about whether it is possible to improve the estimation of the locations based on a specific technology and characteristics of the WSN, or whether it is it possible to meet the requirements demanded by a specific application of localization. The CRLB responds to these types of questions and provides a minimum bound for the variance attainable of any unbiased estimator. An example of the CRLB computation with wsnLocalize is illustrated in Section 4.

### 2.3. Potential Applications 

The IoT will seamlessly integrate a large number of heterogeneous devices [12]. If the localization task can be implemented as described in Figure 2, innovative applications can be developed in different areas. As already mentioned, within the context of industrial safety and manufacturing environments, the development of WSNs in closed spaces, such as the mining industry or other types of indoor facilities can increase the safety of workers by their detection inside unsafe areas [3]. In extreme cases, such as collapse situations in mining operations, knowledge of the position of workers can be critical in rescue operations.

In logistics applications, the LoT service can provide new capabilities for variable monitoring and distributed control, which reduces the cost in those applications that are typically managed in a wired way. In warehouses, different strategically distributed sensors can monitor the environmental conditions and provide this information to the installed heat, ventilation, and air conditioning (HVAC) systems. The sensors coupled to mobile equipment can help locate them in case of loss during the inventoried phase, and even execute certain events if the location of the equipment outside the warehouse is detected. Considering purposes of biological research, animal tracking can answer questions about animal behavior and interactions within their own species as well as with other species. Important information can be inferred by means of the estimation of inter-animal distances using cooperative localization methods [6].

The global accuracy of the location system will be decisive, which is a limiting factor in the development of certain applications. Several aspects must be considered, such as the relative positions of the anchor nodes, the general structure of the WSN (uniform or heterogeneous node placement), the node capabilities in terms of ranging accuracy, or the average connectivity of the WSN. The reader may refer to References [30,31] for a detailed explanation about 5G cellular and IoT applications.

## 3. Developed Graphical User Interface: wsnLocalize

**Figure 3:**
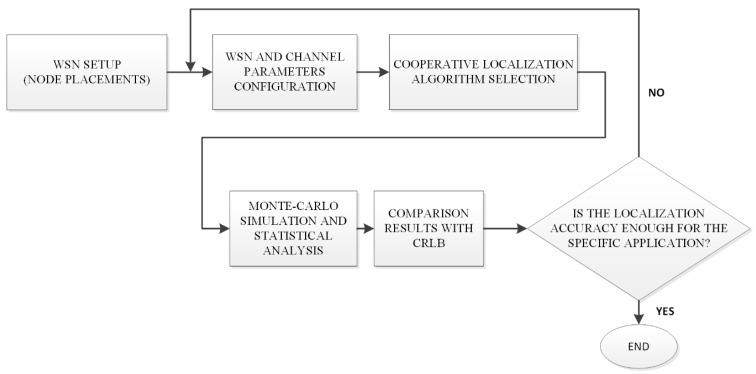
Workflow description of wsnLocalize.

One of the main objectives of wsnLocalize is to provide researchers a flexible framework to interactively evaluate cooperative localization algorithms. The interactivity provided with the tool as well as the capabilities in the definition of the measurement graph complement other tools developed with similar purposes found in the literature [6,32]. Next, the main properties of the developed tool are explained in this section. The tool is available at: http://www.uco.es/grupos/prinia/marioruz. The different stages of using the tool are shown in the workflow of Figure 3.

**Figure 4:**
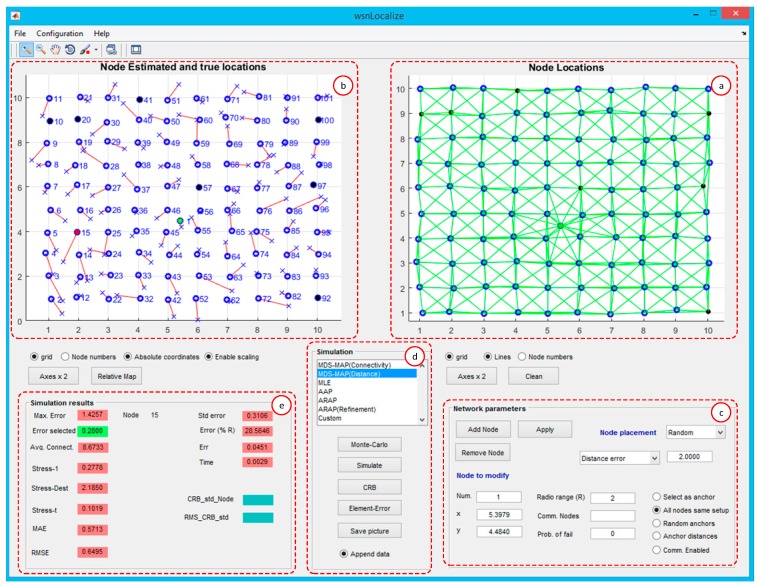
Main window of the developed tool.

Figure 4 shows the main window, which consists of several differentiated areas (a–f). They are described below.

1. **WSN topology (a)**. This plot shows the true node coordinates of the WSN, including the anchor and the blind nodes. The position of each node can be modified either by dragging the node or by editing the x and y coordinates defined in the corresponding text fields. Each time, a node position or a WSN parameter is modified, the graph associated with the modeled WSN is updated and a new simulation is executed.
2. **Node estimated and true locations (b)**. This plot shows the absolute positioning of the nodes and depends on the localization algorithm applied and all the parameters that affect to the pairwise distance estimations (WSN topology, average connectivity, etc.). The plot shows the true and estimated node locations. The *Relative map* push button located below the plot shows the relative map produced such as in MDS techniques. In fact, in the absence of enough anchor nodes, the absolute map cannot be computed.
3. **Network parameters (c)**. This menu allows the configuration of the node graph associated with the WSN. Basically, the user can set up the radio range of connectivity, select the distance error in the pairwise estimated distances, and add/remove blind or anchor nodes. A particular node can be located by specifying its x and y coordinates in the corresponding edit text fields. In addition, the user can set up these features for each particular node in the WSN, including the specification of the probability of failure in the communication. If the select as anchor radio button is enabled, the node specified in the Node to modify (number) text field will be considered as an anchor. Then, its absolute coordinates will be used to transform the relative map to the absolute map (anchor nodes are represented with black points). The distance error added to the true pairwise distances can be specified as a blurred Gaussian noise or be based on a statistical model for RSS or TOA [4]. All nodes with the same setup radio button allow the user to switch between a uniform scenario where all nodes have the same features, or specific features per node. The editable text field Comm. Nodes allows enabling or disabling the possible communication between the node specified in the Node to modify (number) editable text field. On the other hand, if the Random Anchors radio button is enabled, when executing Monte-Carlo simulations, the anchor nodes will be randomly selected in each iteration. The Node placement pop-up menu allows us to automatically generate different WSN topologies, such as random, square, and C-shaped. In all of them, it is possible to specify the number of blind and anchor nodes.
4. **Simulation (d)**. This section allows us to perform simulations of localization algorithms applied to the WSN graph model specified in Section (b). The Simulate button executes one iteration of the specified localization algorithm in the list box. The Monte-Carlo simulations are carried out with the Monte-Carlo push-button. In this case, wsnLocalize runs a specified number of iterations. The results of each iteration are averaged and displayed in a new window. The CRLB push-button calculates the Cramér-Rao Lower Bound for the WSN.
5. **Simulation results (e)**. This section provides relevant information to quantify the performance of the tested localization algorithm, such as the maximum and average error, the standard deviation error, the average connectivity of the WSN, and the localization error of a concrete node specified by the user. In addition, a set of common cost functions to evaluate the goodness of the algorithms have been implemented, such as the Frobenius Norm, ${\mathrm{Stress}}_{1},$ and functions ${\mathrm{Stress}}_{t}$. The detailed information of these cost functions is included in Appendix A. In addition, the maximum error is calculated and displayed, i.e., the maximum difference between the true and estimated location of a particular node, which is also determined. Other interesting results are also shown in this section, such as the averaged error, the root mean squared error (RMSE), the maximum absolute error (MAE), and the average error relative to the radio range (R). Furthermore, the user can select a specific node and obtain specific information about the localization error.
6. **Localization algorithm (f)**. In this section, the user can select the localization algorithm to be tested. As examples, the current version of wsnLocalize provides algorithms based in the multi-dimensional scaling techniques [19,20] and the As-Rigid-As-Possible-Approach algorithm (ARAP) [7]. Nevertheless, the user can test custom localization algorithms by selecting the custom option. In this case, the tool will construct the relative and absolute maps (given enough anchor nodes) and will evaluate its performance, as previously explained.

Additionally, the user can zoom or pan the different axes, save and load sessions, or generate reports based on Monte-Carlo simulations. By using this option, it is possible to obtain statistical conclusions that describe the behavior of the different localization algorithms, according to the model and network parameters that are being simulated. In each iteration, the generated results are saved and, finally, a set of average parameters are stored in a text file y comma separated values (CSV) format. This feature is exploited in Section 4 (illustrative examples).

In previous sections, the main measurements employed for distance estimation were described (RSS, TOA, AOA), as well as the basic stages for cooperative localization algorithms. In most of the works consulted [7,20,21,24], the authors use a set of network parameters to evaluate the performance of the developed algorithms. The typical parameters are a common connectivity range in the nodes with added noise in the measurement error, which is usually modeled as Gaussian noise and added to the true distance, and a uniform radio propagation model is based in the radio range R. In addition, the localization performance of the algorithms is usually evaluated by means of a global computed localization error, such as the mean error or the average normalized error per sensor. One of the objectives of the presented tool is to make the configuration of the measurement graph as flexible as possible.

The graphical user interface has been structured coherently and user-friendly. The main contributions of wsnLocalize can be summarized in the following features.

- WSN parameters (number of nodes, node connectivity, distance estimates, etc.).
- Monte-Carlo simulation.
- Report generation.
- A probabilistic connectivity model is included with the tool [15]. Thus, the disk graph assumption can be avoided.
- Particular node features can be specified (node range, probability of failure).
- A set of stress functions, which evaluate the performance of the localization algorithms computed (see Appendix A), including the amount of time it takes to run an algorithm, and also consider specific node localization statistics.
- Interactivity: the user can move each node interactively and modify its characteristics (convert it to anchor or blind node, modify its node range, etc.).

## 4. Illustrative Examples

In this section, a set of basic cooperative localization algorithms are simulated to demonstrate the capabilities of the developed tool. Two typical scenarios are evaluated regarding the WSN topologies: (a) uniform node placement with placement error $e_{p}=10\%$, where, as in Reference [19], $e_{p}$ is a random value drawing from a normal distribution $r\cdot e_{p}\cdot N\left(0,1\right)$, assuming r is the unit length and (b) node is randomly placed with a uniform distribution. In both cases, the nodes are placed within a 10r·10r square. Particular node features are considered in a third example, and the probability of failure in the communication of some nodes is taken into account. This shows how the localization accuracy is degraded. In addition, the CRB is executed and compared with one of the executed algorithms. When distance information is available, the results shown in the tables represent the averages over 40 trials carried out in the Monte-Carlo simulation. The distance information is modeled as the true distance blurred with Gaussian noise, as explained in Section 3. The connectivity, i.e., the average number of neighbors, is controlled by specifying the radio range R. Given these two parameters (distances and connectivity ranges per node), the anchor nodes, the tool builds an undirected graph, which represents the WSN. Each time a simulation is run (i.e., one iteration), the mentioned graph is updated and the selected localization algorithm is executed.

### 4.1. Grid Distribution

**Figure 5:**
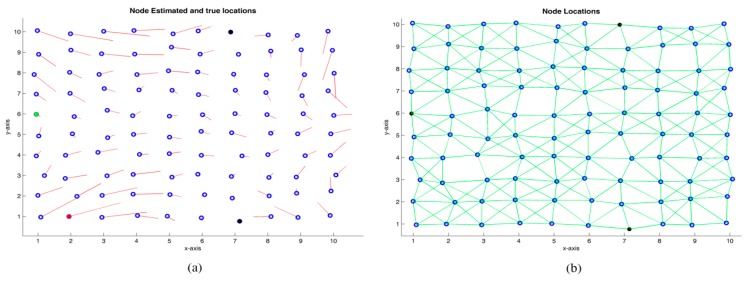
(**a**) Real node positions ( 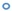 ) and error with respect to the estimated positions (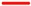), R=1.5r. (**b**) Real positions of the nodes and graph associated with the WSN.

In this example, 100 nodes are placed uniformly on a 10r·10r grid, where r is assumed to be the unit length. For the placement of the nodes, a zero mean Gaussian noise of 10% has been added to the node original positions. The undirected graph, which represents the WSN, is shown in Figure 4b, where edges (green lines) represent the existence of connectivity between a pair of nodes and, thus, a measured distance based on TOA, AOA, RSS, or any combination of them. Conversely, if there is no direct connectivity between a pair of nodes, the distance is estimated by the specific cooperative localization algorithm based on the known estimated distances. In this example, three anchor nodes have been chosen (colored in black) and connectivities of R=1.5 r, 2.5 r, and 3 r are simulated. Monte-Carlo simulations were performed, considering the algorithms MDS-MAP(C), MDS-MAP(P), ARAP, and ARAP+ref (with refining stage) algorithms. For illustrative purposes, Figure 5 shows the estimated positions with the algorithm MDS-MAP(C) and R=1.5 r. With this setup, an average connectivity of 6.08 is obtained. An average error in the node positioning of 0.5564r and a maximum error of 1.7061r are obtained. If r=1 m in the current example, it corresponds to a distribution of nodes in a grid area of 10 m×10 m with mean and maximum errors of 0.5564 and 1.7061 m, respectively.

**Figure 6:**
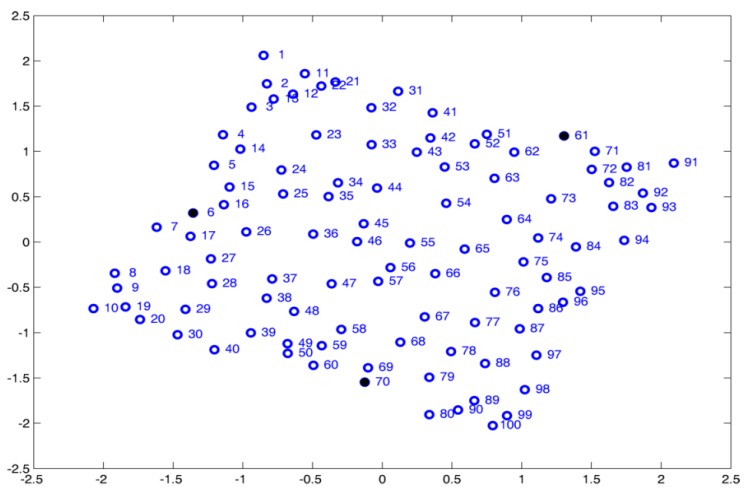
Relative map obtained with MDS-MAP(C) algorithm. R=1.5r.

Figure 6 shows the relative map obtained by the MDS-MAP(C) algorithm. Although this map does not provide absolute coordinates, it still provides useful information. If there are no anchor nodes, relative information may be all that is obtainable [19].

Table 1 shows the statistical parameters obtained in the simulation for a set of algorithms. In each simulation, 40 iterations were performed and the mean values for each of the parameters shown were obtained, using the same anchor nodes for all the iterations. The table shows the name of the algorithm, the added noise in the measured distances, $e_{r}$, expressed as a percentage, the average connectivity in the WSN, C, the average mean error in the localization with respect to the node range, Err(%R), the maximum and the mean error averaged in all the iterations, *Max. Error*, and *Avg. Error*, the standard deviation of the mean error obtained in the positioning of each node (the smaller this parameter, the error in the positioning of each node is more uniform or similar), *Std*(*Error*), the normalized error, Err, the stress function normalized to distances, $Stress_{1}$, and the normalized stress function to distance and invariant to the scaling factor, $Stress_{t}$.

The mean squared error per element of the estimated distance matrix $\tilde{D}$ with respect to D is also included, $Stress_{\tilde{D}}$. This parameter measures the reliability of the input data. Lastly, the calculation time per iteration is also included, *t*(*s*). This parameter must be taken as a relative measure due to its dependence on the speed of the computer where simulations are executed.

It is important to note that, for the ARAP algorithms, scaling transformations have not been considered in the construction of the global map, since the authors based this algorithm on rigid transformations [7]. In fact, it can be verified that when enabling the option Enable scaling in wsnLocalize, the results with ARAP are very similar. Conversely, by disabling this option, the localization performance of MDS-MAP(C) can often yield poor results.

#### Result Analysis of Grid Distribution

From Table 1, several conclusions can be obtained. In the MDS-MAP(C) algorithm, a substantial difference is observed with respect to the functions ${\mathrm{Stress}}_{1}$ and ${\mathrm{Stress}}_{t}$, with the former being larger. This indicates that, if scaling operations are allowed to get the absolute locations, the global positioning accuracy is improved. The positions obtained with the MDS-MAP(C) algorithm are less accurate compared to those obtained with MDS-MAP(D) with $e_{r}=5\%$. Similar results are obtained by their authors [19,20]. The reconstruction of the distance matrix $\tilde{D}$ is the same for the MDS-MAP(D) and ARAP algorithms. This means that the quality of the input data (i.e., estimated distances) for the two compared algorithms is similar. Thus, the performance comparison between the localization algorithms can be considered fair. The average error obtained and the stress functions are lower for ARAP, specifically at low connectivity values, where the mean error is approximately 65% higher for MDS-MAP(D). Nevertheless, as the connectivity increases, this difference decreases. In addition, the calculation time required for the ARAP algorithm increases considerably, reaching three orders of magnitude higher than the MDS-MAP variants. 

**Figure 7:**
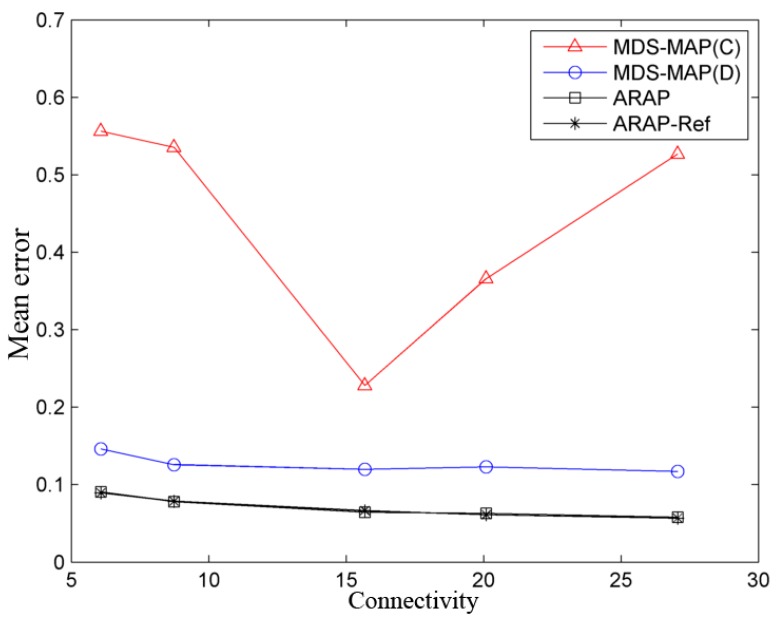
Mean error as a function of the mean connectivity in the WSN, $e_{r}=5\%$, and three anchor nodes.

The mean error of the estimated positions is shown in Figure 7. This error has been obtained as a function of the WSN connectivity, according to the applied algorithm. As can be observed from the figure, prior knowledge of pairwise distances yields an average error smaller than for the case of only connectivity (MDS-MAP(C)). In addition, for the latter case, greater connectivity does not necessarily imply an improvement in estimating node localizations. This situation occurs in those algorithms that use only connectivity as input information, such as MDS-MAP(C). The estimated Euclidean distances are less accurate if the node coverage (radio range) is very high, which implies a higher average connectivity. This can be understood in the following way, if a pair of nodes are located very far from each other and can communicate, they will have a direct connection in the measurement graph, and, therefore, the same connectivity value as those that are closer. In fact, given the maximum connectivity value, i.e., 99 for a WSN of 100 nodes, which indicates that each node has a direct connection with the rest of the nodes, the error with MDS-MAP(C) is increased to 7.98*r*. Given a lower WSN connectivity, the estimated pairwise distances would be proportional to the number of hops, i.e., the smaller number of nodes (shortest path) that are needed to communicate with each other.

This result is not clear in the works carried out by Shang et al. [19,20,21], who are pioneers in applying MDS techniques for localization purposes. When the connectivity of the WSN is increased (i.e., the node connectivity is greater), in these works, the err(%R) is used as a measure of the error, which normalizes the error with respect to the communication range, R, expressing it as a percent. This implies that an err(%R) of 50% is equivalent to an error of 50% of the node range, which can lead to confusion, since, for larger connectivity values and a similar value of err(%R), the absolute error is greater. For this reason, wsnLocalize includes different measurements for evaluating the algorithm performance, such as the absolute average error, or the average error normalized per node, Err, (see Appendix A). 

On the other hand, when a priori pairwise distances can be estimated, as the connectivity increases, and the average error tends to decrease. The approximation to Euclidean distances is improved, especially if the node distribution follows a grid topology [19]. However, given a grid distribution, from approximately *C* = 15, the decrease in the error is almost zero, as shown in Figure 7. It is also observed that the performance of ARAP and ARAP-Ref is practically identical, as stated by their authors [7]. Since the refining stage has practically no effect on the improvement of the estimation of the positions, this provides a large increase in computational complexity in return for a very small increase in performance. In the following examples, only ARAP is considered.

**Figure 8:**
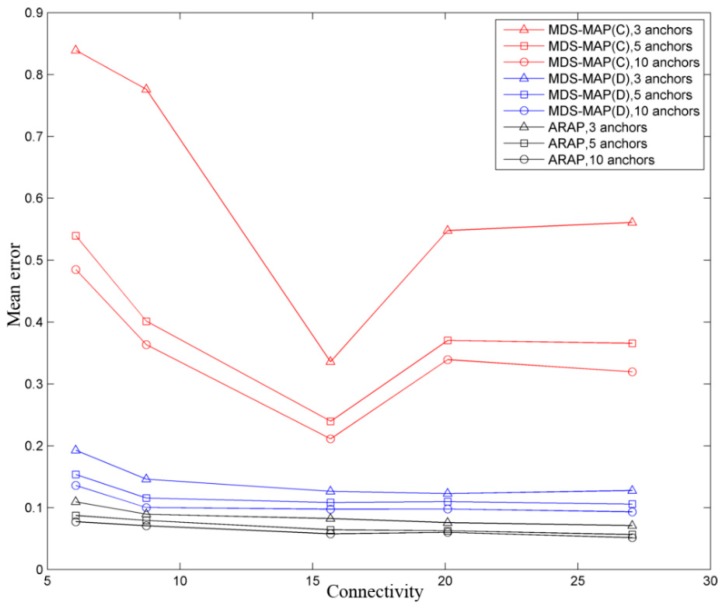
Mean error as a function of the anchor nodes and connectivity in a grid WSN topology.

Lastly, Figure 8 shows the simulation results for the same WSN. However, in this case, the anchor nodes were modified in each iteration. For example, for MDS-MAP(C) with five anchors nodes, 40 iterations have been performed, and the anchor nodes were randomly chosen in each of iteration. A similar process has been done for the other algorithms. As shown in the figure, when the number of anchor nodes is higher, the estimation of the positioning improves at some portion. However, this improvement is less noticeable in the range of five to 10 than three to five. Similar results are obtained in [20,21].

### 4.2. Random Node Distribution

**Figure 9:**
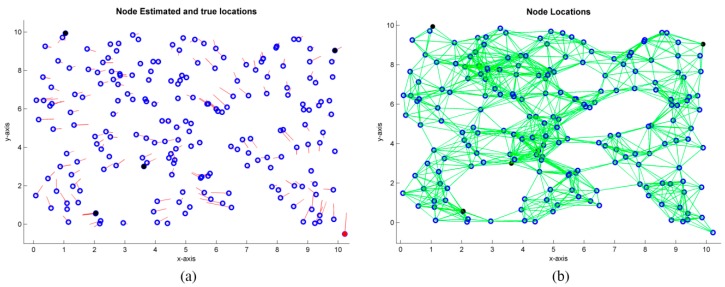
200 nodes randomly distributed. (**a**) True node positions (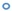) and error with respect to the estimated node positions (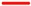). (**b**) True node positions and associated graph to the WSN with R=1.5r.

This example shows how noise added in the pairwise distances impacts the final accuracy of the node position. In this simulation, the sensor nodes are assumed to be randomly distributed. Figure 9 shows 200 nodes in a 10r·10r square. Similar reports, as shown in Table 1, can be obtained with wsnLocalize. In this set of simulations, the performance of MDS-MAP(D) and ARAP algorithms is studied when distances are disturbed with zero mean Gaussian noise of 2%, 5%, 10%, 15%, and 20%. Four anchor nodes are used and a radio range R=1.2r, which, in the case of this example, provides a connectivity value of C=7.98.

**Figure 10:**
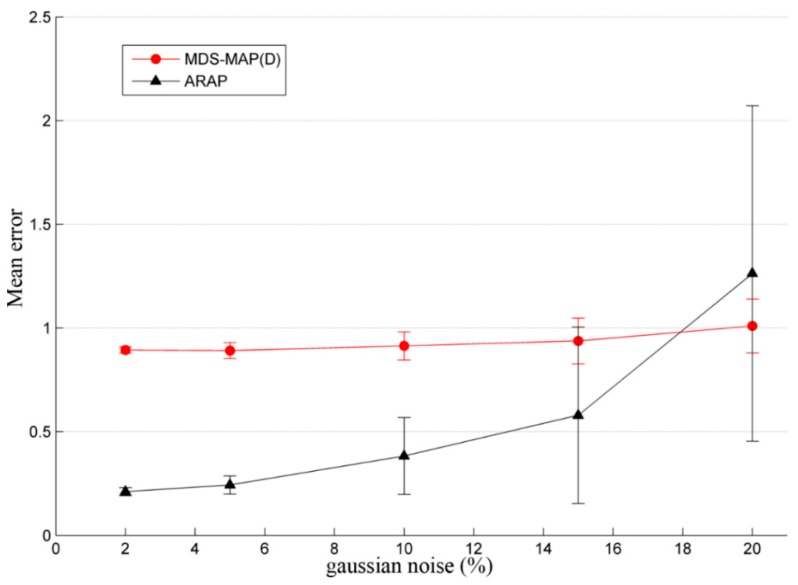
Mean error as a function of the added noise in the pairwise distances. Vertical bars indicate the sum and difference of the standard deviation of the mean error, i.e., the dispersion of the mean error considering all the iterations in the Monte-Carlo simulation.

Figure 10 shows the evolution of the mean error (measured in relative units, as in the previous example) since the added noise is increased. As shown in the figure, it can be observed that MDS-MAP(D) slightly increases the average error obtained and its standard deviation. Conversely, the same does not happen with the ARAP algorithm. While, for low added noise, the mean error is lower than the one obtained with MDS-MAP(D). A significant degradation is appreciated for $e_{r}>15\%$, which reaches a higher average error than the obtained with MDS-MAP(D) from $e_{r}=20\%$. Furthermore, from a noise value of $e_{r}=10\%$, the standard deviation of the average error in the iterations increases considerably. The dispersion of the error is measured by the vertical bars shown in Figure 10, i.e., these bars indicate the standard deviation of the mean error, providing a parameter of the algorithm sensitivity of the algorithm against noise in the estimated distances.

#### Result Analysis of Random Distribution

Figure 9 shows the specific case of $e_{r}=10\%$, R=1.5r, with a connectivity level of C=12.09, where positioning is shown in one of the iterations using MDS-MAP(D). For the latter case, the estimation of the positions is improved with respect to the previous case (R=1.2r). This is expected, since the increase in connectivity allows a better estimation of the pairwise distances. Table 2 shows the statistical data obtained with wsnLocalize and MDS-MAP(D) with different noise levels in the estimated distances. As seen in Table 2, an increase in the connectivity range causes a decrease in the mean error of at least a half, and the maximum error is reduced at least to the third part.

Given the anchor node placements shown in Figure 9, the statistical results obtained by simulation indicate that MDS-MAP(D) has a similar behavior in terms of performance despite the fact $e_{r}$ is increased. In contrast, the ARAP algorithm produces better results than MDS-MAP(D) given estimated pairwise distances with $e_{r}<18\%$. Nevertheless, when $e_{r}$ exceeds 15%, the performance of ARAP is significantly degraded. In other words, when the reliability of the estimated distance matrix $\tilde{D}$ is poor, MDS-MAP(D) provides better results than ARAP. Furthermore, as mentioned previously and shown in Table 1, ARAP requires more computational time.

The MDS-MAP algorithms provide better results in WSNs with grid distribution in comparison with WSNs with random distribution. This result can be observed by comparing Table 1 and Table 2, where maximum, average, and normalized errors of larger magnitude occur in the random distribution. Coherent results are obtained, as expected, in the $Stress_{1}$ and $Stress_{t}$ functions, i.e., for equal connectivity and noise conditions, the grid distribution provides more precise localization results regardless of the algorithm applied. On the other hand, the performance of ARAP is assumed to be superior to MDS-MAP(D) algorithms in Reference [7]. However, in the mentioned work, the authors considered an added zero mean Gaussian noise in the estimated distances up to 10%. As shown in Figure 10, the average error obtained with ARAP is lower than with MDS-MAP(D) for $e_{r}=10\%$. As $e_{r}$ increases, ARAP loses robustness and becomes more sensitive to the accuracy in the distance estimations, to such an extent that the average error obtained is greater than the one obtained when applying MDS-MAP(D). As seen in Figure 10, this occurs from approximately $e_{r}=18\%$.

Similar results were obtained for a WSN of 50 nodes with a grid distribution, with five anchor nodes, $e_{r}=20\%$, R=2, and C=8.4. In this case, the maximum error, the average error, and the standard deviation are greater in ARAP than MDS-MAP(D). However, if $e_{r}=10\%$, ARAP is more accurate than MDS-MAP(D). Thus, it seems that the behavior of ARAP is degraded more than MDS-MAP(D) when the pairwise distance error (of Gaussian type) is greater than 20%. One reason of this result is that ARAP is based on “stitching” together local structures in the sensor graph in an as-rigid-as-possible manner. Specifically, the algorithm looks for reference patches constructed by means of reference triangles, which are obtained if the distances between three nodes are given [7]. If formed triangles contain high errors in the estimated distances, the union between them by means of rigid transformations does not represent adequately the true positions of the nodes.

In a real case, distance errors can be greater than 10%, especially in pairwise distance estimates with low connectivity and based on RSSI. Regarding the absolute map transformation, in [7], all nodes are supposed to be an anchor. In the example, this assumption is not made, and four anchor nodes are supposed instead. As a main conclusion, it has been verified, as expected, that noise in the measured distances affects the goodness of the solutions provided by the positioning algorithms. Nevertheless, for high noise levels $\left(e_{r}>20\%\right)$, MDS-MAP(D), which is an algorithm with lower computational cost than ARAP, provides better localization performance results.

### 4.3. Non-Disk-Graph Assumption and Probabilistic Model in Connectivity

In the two previous examples, all nodes were assumed with the same radio range. Furthermore, a disk graph model was supposed, i.e., two nodes are connected if (and only if) the distance between them is less than a given distance d. This model is typically used for testing localization algorithms [4,7,19,20,21], since a sensor typically can communicate only with its local neighborhood, and the connectivity tends to be local. Nevertheless, this approach is not realistic in practice. Although having the WSN model as simple as possible is interesting, if certain restrictions are not considered, such as the fact that a set of nodes cannot communicate with each other due to the presence of obstacles or shadowing, the performance indices may be overoptimistic for the considered scenario. Even considering these impairments, it can happen that, on certain occasions, the nodes cannot communicate due to another cause, e.g., due to a fault, interferences in the environment, etc. The developed tool allows us to assign particular failure probabilities for each node in the WSN, in addition to specifying those pairs of nodes that cannot communicate with each other even if their distance is less than the connectivity range. This last situation can occur in case of non-line-of-sight (NLOS), where the path of propagation is obscured by obstacles, which makes it difficult for the radio signal to pass through, and, thus, communicate.

**Figure 11:**
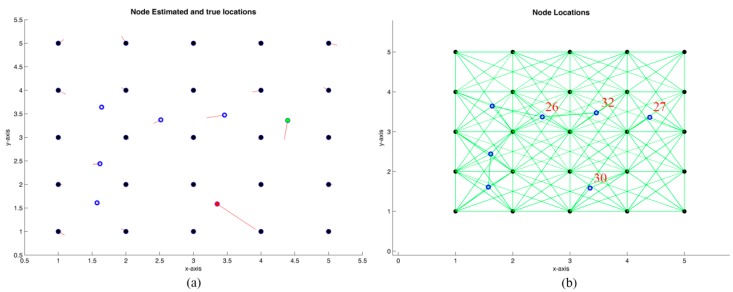
Grid distribution with 25 anchors nodes and seven blind nodes. (**a**) Node locations in one of the iterations. (**b**) WSN graph.

Figure 11 shows a simple example, with 25 anchor nodes and seven blind nodes. Table 3 shows the simulation results after 40 iterations, considering two cases. In the first one, a connectivity range R=2.5r is assumed for all the anchor nodes, and a noise in the distances of $e_{r}=5\%$. However, in this case, the radio range of the blind nodes is considered shorter. This situation could be assimilated to a controlled environment, where the anchor nodes have superior communication capabilities and the blind nodes are transmitters embedded in or attached to workers [3].

The second case considers a certain failure probability of communication (second row of Table 3), where the blind nodes have a radio range $R_{p}=1\;m$ and failure probabilities of 0.1, 0.5, 0.2, and 0.3 are also considered for the nodes in Figure 11 labeled with the numbers 27, 30, 32, and 26, and a noise in the distance reading $e_{r}=10\%$.

As seen in Table 3, the fact of considering particular node features and specific connectivity ranges decreases the localization accuracy. This is logical, since including probabilities of failure cause a degradation in the accuracy of the estimated distances, as can be observed in the functions $Stress_{t}$ and $Stress_{\tilde{D}}$ in Table 3. Considering these characteristics, the statistical values obtained with wsnLocalize show an average maximum error five times greater for the non-uniform case, and an average error of approximately double.

#### CRLB Example

**Figure 12:**
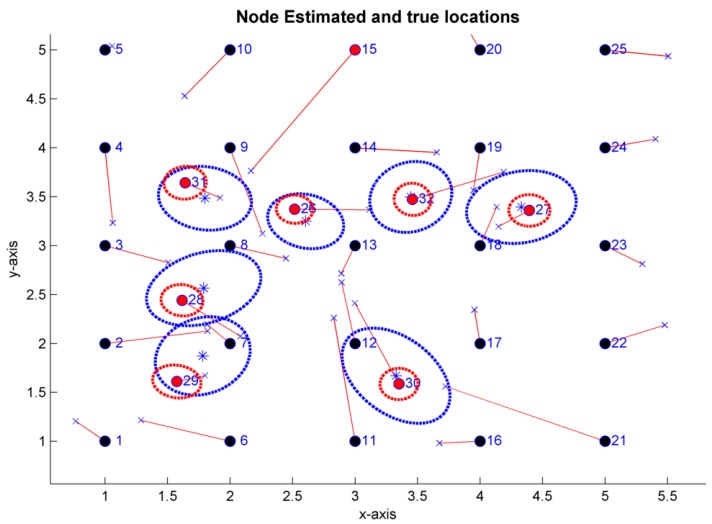
Estimator 1−σ uncertainty ellipses (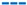) and CRLB-TOA on the 1−σ uncertainty ellipses (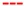). R=1.5 m,std(TOA)=0.5 m.

As previously mentioned, the CRLB has been implemented in wsnLocalize. This feature provides the capacity to the tool to analyze the best an estimator can possibly do for a given WSN topology and a given set of measurements. As an application example, the distance estimates are assumed to be based in TOA techniques and perturbed with zero mean Gaussian noise. Considering a standard deviation error of 0.5 m for TOA measurements, an average error of 0.2308 m is obtained. The averaged standard deviation of the CRLB is calculated averaging the localization variances obtained in the blind nodes and calculating the square root of the result. In this case, a connectivity range of R=2.5 m is considered. By means of Monte-Carlo simulations and running MDS-MAP(D), a standard deviation error of 0.5449 m in the localization of the blind nodes is obtained. Figure 12 shows the uncertainty ellipses for each blind node, obtained by means of the CRLB (red color) and by simulation of the MDS-MAP(D) algorithm (blue color), as well as both calculated within an interval of 1−σ, where σ is the standard deviation obtained in the localization of each blind node in particular. In the figure, the red lines show the positioning of the nodes in one of the iterations of the Monte-Carlo simulation, of which only those of the blind nodes are of interest, since the positions of the anchor nodes are known (although MDS-MAP algorithm yields node coordinates considering all the nodes of the network). The average of the localization estimates of the blind nodes has been represented by blue asterisks.

For this case, the CRLB obtained shows that a better estimator for the given WSN characteristics is possible. The corresponding bound for the standard deviation of localization error when the WSN connectivity is maximum is 0.1843 m, which is approximately three times smaller than the standard deviation obtained with MDS-MAP(D).

## 5. Conclusions

An interactive tool, named wsnLocalize, designed for the simulation of cooperative localization techniques has been developed. The tool is intended to compare and evaluate such methods, providing researchers and designers a fast way to measure the performance of different localization algorithms considering specific network topologies. The interactivity provided allows the user to instantaneously observe the effects on the localization performance by simply changing a node position, by dragging it to a new one. In the same way, a different set of parameters can be changed, such as the noise added to the distance measurements, the node range, or the probability of failure. WsnLocalize exploits the Monte-Carlo method, which allows the generation of statistical reports for a concrete WSN topology and a specific localization algorithm. One of the main contributions of the developed tool is its capability to consider independent characteristics for each node in the WSN, such as the node range connectivity. This feature allows the avoidance of the typical “disk graph model,” which is usually applied when localization algorithms are tested. It has been shown how adding specific features to the nodes, considering their probability of failure in the communication and a particular connectivity range, among other parameters, affect the performance of localization algorithms.

From the authors’ perspective, real time positioning systems based on radio frequency will enhance provide new capabilities in a wide variety of applications. The integration of LoT in the IoT paradigm will provide the localization service in new scenarios. Additionally, the robustness of the localization system must be considered and tested. Parameters such as the accuracy of the positioning in different conditions, immunity to multipath fading, power consumption of the sensors, and range or latency, are critical application-specific factors to be considered. 

As future directions of research, it is desirable to continue improving the capabilities of wsnLocalize, including more complex propagation models for the distance estimates and adding more cooperative localization algorithms, such as SPAWN, dwMDS, or HSPAWN. Another interesting topic is the distance reconstruction from incomplete distance information. The implementation and testing of provable accurate reconstruction methods in conjunction with a localization algorithm is also of particular interest.

## Figures and Tables

**Table 1:** Monte-Carlo simulations for 100 nodes uniformly distributed in a 10r·10r grid. Three anchor nodes, $e_{p}=10\%$.

Algorithm	*R*	$e_{r}$	*C*	*Err(%R)*	*Max. Error*	*Avg. Error*	*Std(Error)*	*Err.*	$Stress_{1}$	$Stress_{t}$	$Stress_{\tilde{D}}$	*t*(s)
MDS-MAP(C)	1.5	-	6.08	37.0924	1.7061	0.5564	0.3607	0.0439	0.1395	0.0879	0.4428	0.0087
MDS-MAP(C)	2	-	8.74	26.7743	1.6048	0.5355	0.3056	0.0422	0.3060	0.0625	2.9580	0.0069
MDS-MAP(C)	2.5	-	15.68	9.1161	0.5433	0.2279	0.1161	0.0180	0.5026	0.0372	7.7092	0.0087
MDS-MAP(C)	3	-	20.1	12.1917	1.1245	0.3658	0.2236	0.0289	0.5866	0.0620	10.3735	0.0092
MDS-MAP(C)	3.5	-	27.06	15.0447	1.3753	0.5266	0.2677	0.0415	0.6428	0.0582	12.6875	0.0087
MDS-MAP(D)	1.5	5	6.08	9.9766	0.4016	0.1496	0.0846	0.0118	0.0509	0.0248	0.0979	0.0071
MDS-MAP(D) ^1^	1.5	5	6.08	12.14	0.4597	0.1821	0.0969	0.0144	0.0510	0.0247	0.0987	0.0073
MDS-MAP(D)	2	5	8.74	6.2893	0.3428	0.1258	0.0689	0.0099	0.0256	0.0184	0.0310	0.0072
MDS-MAP(D)	2.5	5	15.68	4.7834	0.3277	0.1196	0.0648	0.0094	0.0251	0.0178	0.0227	0.0071
MDS-MAP(D)	3	5	20.1	4.0988	0.3349	0.1230	0.0677	0.0097	0.0341	0.0180	0.0347	0.0073
MDS-MAP(D)	3.5	5	27.06	3.3372	0.3108	0.1168	0.0623	0.0092	0.0431	0.0173	0.0558	0.0072
ARAP	1.5	5	6.08	6.0295	0.2481	0.0904	0.0487	0.0071	0.0145	0.0140	0.1001	0.0648
ARAP	2	5	8.74	3.8995	0.2352	0.0780	0.0432	0.0062	0.0125	0.0122	0.0306	1.5229
ARAP	2.5	5	15.68	2.5655	0.1852	0.0641	0.0351	0.0051	0.0102	0.0100	0.0240	3.4588
ARAP	3	5	20.1	2.0914	0.1924	0.0627	0.0360	0.0049	0.0101	0.0098	0.0352	5.1697
ARAP	3.5	5	27.06	1.6556	0.1577	0.0579	0.0309	0.0046	0.0094	0.0091	0.0554	8.6734
ARAP + ref	1.5	5	6.08	5.9508	0.2296	0.0893	0.0469	0.0070	0.0135	0.1300	0.1002	1.0318
ARAP + ref	2	5	8.74	3.6761	0.1928	0.0735	0.0384	0.0058	0.0121	0.0116	0.0309	1.5599
ARAP + ref	2.5	5	15.68	2.5927	0.1779	0.0648	0.0354	0.0051	0.0098	0.0096	0.0232	3.5110
ARAP + ref	3	5	20.1	2.0284	0.1701	0.0609	0.0336	0.0048	0.0097	0.0094	0.0375	5.2147
ARAP + ref	3.5	5	27.06	1.6206	0.1535	0.0567	0.0299	0.0045	0.0090	0.0089	0.0548	8.7376

^1^ In this case, anchor nodes were randomly modified in each iteration.

**Table 2:** MDS-MAP(D) Monte-Carlo simulations for 200 nodes randomly distributed in a 10r·10r grid. Three anchors nodes, different noise levels in the estimated pairwise distances ($e_{r}$).

*R*	$e_{r}$	*C*	*Err(%R)*	*Max. Error*	*Avg. Error*	*Std(Error)*	*Err.*	$Stress_{1}$	$Stress_{t}$	$Stress_{\tilde{D}}$
1.2	2	7.98	74.4760	3.5111	0.8937	0.6045	0.0683	0.1434	0.1217	1.1725
1.2	5	7.98	74.2523	3.5053	0.8910	0.6002	0.0681	0.1381	0.1233	1.0728
1.2	10	7.98	76.1126	3.5181	0.9134	0.6116	0.0698	0.1342	0.1300	0.8996
1.2	15	7.98	78.1152	3.5794	0.9374	0.6290	0.0716	0.1383	0.1373	0.7591
1.2	20	7.98	84.1155	3.7922	1.0094	0.6806	0.0771	0.1615	0.1519	0.7544
1.5	2	12.09	20.1486	1.0665	0.3022	0.1861	0.0217	0.0642	0.0479	0.1884
1.5	5	12.09	21.0064	1.0631	0.3151	0.1871	0.0227	0.0563	0.0511	0.1460
1.5	15	12.09	28.7786	1.3252	0.4317	0.2395	0.0311	0.1062	0.0711	0.2849
1.5	20	12.09	34.4011	1.6036	0.5160	0.2885	0.0371	0.1691	0.0862	0.7217

**Table 3:** Uniform vs non-uniform environment of the example in Figure 9.

Case	C	*Max. Error*	*Mean Error*	*Err*	$Stress_{t}$	$Stress_{\tilde{D}}$
Uniform	17	0.1467	0.0590	0.0104	0.0248	0.0093
Non-uniform	11.1250	0.7124	0.1015	0.0179	0.0617	0.0546

## Appendix A

In addition to typical statistical measures (maximum error, standard deviation error, mean error, root mean squared error, etc.), Table A1 in this appendix contains the stress functions that have been implemented in wsnLocalize for the evaluation of the performance of cooperative localization algorithms. These cost functions have been included since most of the consulted references employ at least one of them to compare the cooperative localization algorithms proposed by different authors [4,7,19,20,23,33].

**Table A1:** Stress functions implemented in wsnLocalize.

Function Name	Equation
$Stress_{1}$	$Stress_{1}\left(x_{1},\ldots ,x_{n}\right)=\sqrt{\frac{\sum_{\left(i,j\right)\in E}^{}{\left(\|x_{i}-x_{j}\|-d_{i,j}\right)}^{2}}{\sum_{i,j}d_{i,j}^{2}}}$
$Stress_{t}$	$Stress_{t}\left(t\cdot x_{1},\ldots ,t\cdot x_{n}\right)=\sqrt{1-\frac{\sum_{\left(i,j\right)\in E}^{}{\left(\|x_{i}-x_{j}\|\cdot D_{i,j}\right)}^{2}}{\sum_{i,j}D^{2}\cdot \sum_{i,j}\|x_{i}-x_{j}\|{}^{2}}}$
Average normalized error per node	$Err=\frac{\sum_{i=1}^{N}\|x_{i}-{\tilde{x}}_{i}\|}{n\cdot max\left(D_{i,j}\right)}$
Normalized error to radio range (%)	$Err\;\left(\%\right)=\frac{\sum_{i=1}^{N}\|x_{i}-{\tilde{x}}_{i}\|}{N}\cdot 100$
Frobenius norm	$Frob=\|D-\tilde{D}{\|}_{F}^{2}/N^{2}$
